# Association between health behaviours and depression: findings from a national cross-sectional study in South Korea

**DOI:** 10.1186/s12888-020-02628-7

**Published:** 2020-05-14

**Authors:** Bich Na Jang, Hyeon Ji Lee, Jae Hong Joo, Eun-Cheol Park, Sung-In Jang

**Affiliations:** 1 Department of Public Health, Graduate School, Yonsei University, Seoul, Republic of Korea; 2 Institute of Health Services Research, Yonsei University, Seoul, Republic of Korea; 3 Department of Preventive Medicine and Institute of Health Services Research, Yonsei University College of Medicine, 50 Yonsei-ro, Seodaemun-gu, Seoul, 03722 South Korea

**Keywords:** Health behaviour, Depression, Walking, Smoking, Alcohol drinking

## Abstract

**Background:**

Depression is a leading cause of disability, and it has been reported that more than 264 million people worldwide have depression. The causes of depression may be numerous, and physical health has also been linked to depression. Therefore, the aim of this study was to determine the effect of health behaviours on depression.

**Methods:**

This study used the data of 224,868 participants from the Community Health Survey, conducted in 2017. We defined health behaviours by combining three variables: no smoking, not belonging to high-risk drinking group, and walking frequently. Depression was measured using the Patient Health Questionnaire-9. Logistic regression was used to examine the association between health behaviours and depression.

**Results:**

Both men and women who did not practise health behaviours were more likely to experience depressive symptoms than those who did (men, odds ratio (OR): 1.48, 95% confidence interval (CI): 1.31–1.68; women, OR: 1.42, 95% CI: 1.32–1.53). Not walking frequently had the strongest association with depression in men and the risk of depression was the highest in women who smoked. Participants who did not practise any health behaviours were the most likely to have depressive symptoms (men, OR: 1.69, 95% CI: 1.38–2.07; women, OR: 3.08, 95% CI: 2.27–4.19).

**Conclusion:**

Our study found that lack of health behaviours is significantly associated with depression. Furthermore, the most influential factor of health behaviours in depression was different for men and women. It is necessary to manage depression through interventional methods customised to gender characteristics. Additionally, national-level policies are needed to encourage steps to improve personal lifestyles, including practising health behaviours.

## Background

Suicide is a serious public health problem worldwide. South Korea is the nation with the major suicide rate (24.6 deaths per 100,000 people) among the Organization for Economic Co-operation and Development (OECD) nations [1]. Depression is one of the most common mental health disorders. It is a leading cause of disability and suicide and it has been reported that more than 264 million people worldwide have depression [2]. Depression also causes other unfavorable outcomes in terms of role functioning, quality of life, and many long-lasting physical health problems [3].

Poor physical health is closely related with depression. It is a well-known fact that people with chronic diseases have a greater tendency to be depressed than healthy people. Cardiovascular diseases such as acute myocardial infarction and stroke and cancer are associated with depression and people who were not depressed prior to having such diseases would develop depression [4–8].

Health behaviour is any activity undertaken by an individual for the purpose of maintaining health and preventing illness [9]. For example, prior research has shown that a variety of health behaviours, including physical activity, are related to depression [10]. Another study proved that an inverse relationship existed between the amount of leisure-time physical activity and symptoms of depression [11]. In addition, people who had depression were more likely to enjoy smoking and drinking [12]. A longitudinal study found that those who had never smoked before the onset of depressive symptoms tended to be more dependent on tobacco than those who smoked before [13]. Such a tendency would eventually lead to poor physical health.

It is important to implement early interventions for depression to get better outcomes [14]. A few countries have made impressive progress in establishing and publishing mental health data for qualifying for care [15]. In South Korea, a substantial number of people have depression [16]; therefore, the government is progressing care for mental disorders from primary to tertiary [17]. However, a national strategic approach in mental health care is still lacking among OECD nations. While localised efforts have been undertaken to improve the collection of indicators of quality of mental health care, such steps are not carried out at the national level [15].

Interventions for reducing depressive symptoms are various and extensive [18]. To find out the most effective way, we should understand the association between health behaviours and depression. As mentioned above, several studies have found an association between health behaviours and depression. However, there is limited research on the association between a combination of health behaviours and depression.

We formed a hypothesis that the fewer the health behaviours practised, the higher the risk of depression. Thus, this study’s main objective is to determine the most influential health behaviours among people with depression and those without. Moreover, previous studies have indicated gender differences in depression [19, 20]. Therefore, our second objective is to investigate the association between health behaviours and depression stratified by gender.

## Methods

### Study population

This study used data from the Korea Community Health Survey (KCHS) conducted in 2017. This survey has been conducted annually by the Korean Centers for Disease Control and Prevention for adults aged 19 or older since 2008 to establish and evaluate regional health plans and standardise the survey performance system to produce comparable regional health statistics [21]. The CHS data used in this study included 201 questions across 18 fields such as health behaviours, physical activities, medical service use, and social environments. The data were not reviewed by an institutional review board on the basis of the Bioethics Act and Article 2 of its enforcement regulations.

**Fig. 1:**
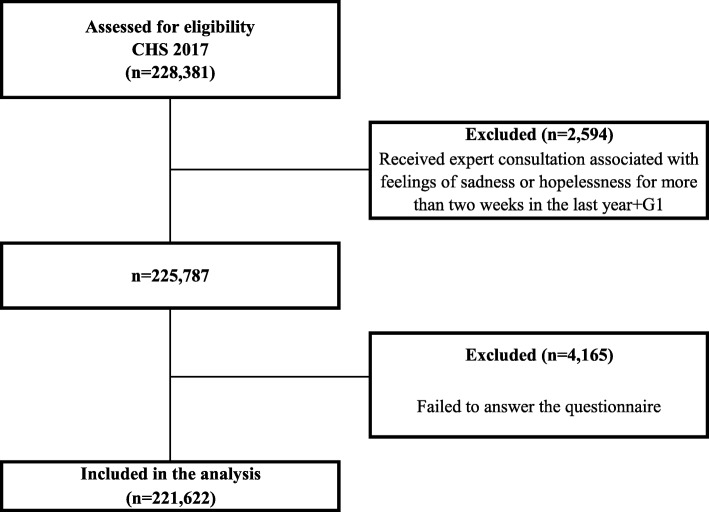
Flow diagram of subject inclusion and exclusion CHS: Community Health Survey

The total population was 228,381 participants; we excluded participants who had received expert consultation associated with sadness or hopelessness for more than two weeks in the previous year (*n* = 2594) to detect new onset of depression. In addition, we omitted those who answered ‘don’t know’ or rejected responses to the questions or had missing data to the questions included in this study (*n* = 4165). Finally, a total of 221,622 participants (99,852 men, 121,770 women) were selected (Fig. 1).

### Variables

To define health behaviours, we combined three variables suggested by the CHS survey: No smoking, not belonging to high-risk drinking group, and walking frequently. No smoking meant not smoking at the time of investigation and those who had ‘0’ pack-year. Pack-year is a method of measuring the amount of cigarettes a person has smoked and calculated by multiplying the number of packs of cigarette smoked per day by the number of years of smoking [22]. We combined these two indicators to assess the exact exposure status of smoking. Not belonging to high-risk drinking group meant being a non-drinker or drinking under five shots (for women) or under seven shots (for men) in a single sitting and having drinks less than one time per week. Walking frequently was defined as walking over 30 min daily more than five days in the last week. Participants who met all three of the above conditions belonged to the practising-health-behaviour group, while those who failed to meet any of the above conditions belonged to the not-practising-health-behaviour group.

The Patient Health Questionnaire-9 (PHQ-9) is a self-administered questionnaire comprising nine questions to evaluate depressive symptoms [23]. For detecting depression, we used the Korean version of PHQ-9, whose validity and reliability has been proven [24]. The score ranges from 0 to 27, and more than 10 points is classified as depression [23]. We divided the participants into two groups according to the score: Have depressive symptoms and no depressive symptoms.

Other covariates were included in the analysis as potential confounding variables: sex, age, marital status, region, occupation category, educational level, household income, body mass index, comorbidity, perceived health status, and perceived stress level. Occupation was categorised according to the Korean version of the Standard Classification of Occupations, based on the International Standard Classification of Occupations by the International Labour Organization. We re-categorised occupation into four categories: White (office work), Pink (sales and service), Blue (agriculture, forestry, fishery, and armed forces occupation), and inoccupation. Comorbidity included hypertension, diabetes mellitus, hyperlipidaemia, and arthritis, and we calculated the number of comorbid diseases that one person had simultaneously.

### Statistical analysis

The covariates were compared using the chi-squared test to confirm association between health behaviours and depression. After adjusting for demographic, socioeconomic. and health-related variables, we used multiple logistic regression analysis to evaluate the association between health behaviours and depression. The results were reported using odds ratios (ORs) and confidence intervals (CIs). Subgroup analysis was also performed stratified by gender and other covariates. In addition, each factor of a health behaviour was analysed through multiple logistic regression analysis to determine which factor was the most relevant to depression. Furthermore, to determine a better-fitting model including all the above-mentioned variables, we examined the Akaike Information Criteria to compare models (Table 1 in the supplementary materials). Differences were considered statistically significant at *p*-values of < 0.05. All statistical analyses were performed using SAS software (version 9.4, SAS Institute, Cary, NC, USA).

## Results

For the purpose of this study, we analysed each variable according to gender. Table 1 shows general characteristics of the study population. Among the 221,622 study participants, 2152 men (2.2%) and 4443 women (3.6%) met the criteria for depression. The number of participants in the not-practising-health-behaviours group was 75,812 men (75.9%) and 78,939 women (64.8%). In the not-practising-health-behaviours group, 1809 (2.4%) of men and 3361 (4.3%) of women had depressive symptoms. On the other hand, 343 (1.4%) of men and 1082 (2.5%) of women who met the criteria for depression were in the practising-health-behaviours group.

**Table 1:** General characteristics of the study population

Variables	Depression													
	Male							Female						
	TOTAL		Yes		No		***P-value***	TOTAL		Yes		No		***P-value***
	N	%	N	%	N	%		N	%	N	%	N	%	
**Total(*N*** **= 221,622)**	99,852	100.0	2152	2.2	97,700	97.8		121,770	100.0	4443	3.6	117,327	96.4	
**Health behaviours**^**a**^							< 0.0001							< 0.0001
Yes	24,040	24.1	343	1.4	23,697	98.6		42,831	35.2	1082	2.5	41,749	97.5	
No	75,812	75.9	1809	2.4	74,003	97.6		78,939	64.8	3361	4.3	75,578	95.7	
**Age (years)**							< 0.0001							< 0.0001
19–29	11,157	11.2	249	2.2	10,908	97.8		12,476	10.2	571	4.6	11,905	95.4	
30–39	13,706	13.7	254	1.9	13,452	98.1		15,405	12.7	406	2.6	14,999	97.4	
40–49	18,051	18.1	232	1.3	17,819	98.7		20,344	16.7	364	1.8	19,980	98.2	
50–59	20,094	20.1	308	1.5	19,786	98.5		23,692	19.5	531	2.2	23,161	97.8	
≥ 60	36,844	36.9	1109	3.0	35,735	97.0		49,853	40.9	2571	5.2	47,282	94.8	
**Marital Status**							< 0.0001							< 0.0001
Living with spouse	72,806	72.9	1252	1.7	71,554	98.3		77,342	63.5	1968	2.5	75,374	97.5	
Living without spouse	27,046	27.1	900	3.3	26,146	96.7		44,428	36.5	2475	5.6	41,953	94.4	
**Region**							0.4589							0.0300
Metropolitan area	29,861	29.9	628	2.1	29,233	97.9		36,647	30.1	1272	3.5	35,375	96.5	
Rural	69,991	70.1	1524	2.2	68,467	97.8		85,123	69.9	3171	3.7	81,952	96.3	
**Occupational categories**^**b**^							< 0.0001							< 0.0001
White	22,866	22.9	220	1.0	22,646	99.0		21,160	17.4	457	2.2	20,703	97.8	
Pink	10,382	10.4	163	1.6	10,219	98.4		18,084	14.9	440	2.4	17,644	97.6	
Blue	42,702	42.8	607	1.4	42,095	98.6		25,984	21.3	683	2.6	25,301	97.4	
Inoccupation	23,902	23.9	1162	4.9	22,740	95.1		56,542	46.4	2863	5.1	53,679	94.9	
**Educational level**							< 0.0001							< 0.0001
Middle school or less	26,477	26.5	985	3.7	25,492	96.3		51,514	42.3	2690	5.2	48,824	94.8	
High school	31,315	31.4	577	1.8	30,738	98.2		31,466	25.8	810	2.6	30,656	97.4	
College or over	42,060	42.1	590	1.4	41,470	98.6		38,790	31.9	943	2.4	37,847	97.6	
**Household income**							< 0.0001							< 0.0001
Low	16,115	16.1	940	5.8	15,175	94.2		28,027	23.0	1978	7.1	26,049	92.9	
Mid-low	34,409	34.5	644	1.9	33,765	98.1		39,101	32.1	1315	3.4	37,786	96.6	
Mid-high	29,230	29.3	346	1.2	28,884	98.8		32,080	26.3	718	2.2	31,362	97.8	
High	20,098	20.1	222	1.1	19,876	98.9		22,562	18.5	432	1.9	22,130	98.1	
**Obesity Status (BMI)**^**c**^							< 0.0001							< 0.0001
Underweight and Normal range	38,382	38.4	1143	3.0	37,239	97.0		69,975	57.5	2689	3.8	67,286	96.2	
Overweight	28,040	28.1	430	1.5	27,610	98.5		25,518	21.0	740	2.9	24,778	97.1	
Obese	33,430	33.5	579	1.7	32,851	98.3		26,277	21.6	1014	3.9	25,263	96.1	
**The number of chronic diseases**^**d**^							< 0.0001							< 0.0001
0	59,400	59.5	968	1.6	58,432	98.4		66,411	54.5	1685	2.5	64,726	97.5	
1	23,901	23.9	581	2.4	23,320	97.6		27,133	22.3	1087	4.0	26,046	96.0	
≥ 2	16,551	16.6	603	3.6	15,948	96.4		28,226	23.2	1671	5.9	26,555	94.1	
**Perceived health status**							< 0.0001							< 0.0001
Good	42,332	42.4	209	0.5	42,123	99.5		39,527	32.5	419	1.1	39,108	98.9	
Bad	57,520	57.6	1943	3.4	55,577	96.6		82,243	67.5	4024	4.9	78,219	95.1	
**Perceived stress**							< 0.0001							< 0.0001
Substantial	22,530	22.6	1531	6.8	20,999	93.2		28,889	23.7	3121	10.8	25,768	89.2	
Less	77,322	77.4	621	0.8	76,701	99.2		92,881	76.3	1322	1.4	91,559	98.6	

^a^Those classified as practising-health-behaviours group met all three conditions: not smoking (or 0 pack-year), not belonging to high-risk drinking group and walking for 30 min over 5 days per week

^b^Three groups (white, pink, blue) based on the International Standard Classification Occupations codes. Inoccupation group includes housewives

^c^*BMI* Body mass index/obesity status defined by BMI based on the 2018 Clinical Practice Guidelines for Overweight and Obesity in Korea

^d^Chronic disease was defined as diagnosed diseases: hypertension, diabetes mellitus, hyperlipidaemia and arthritis. The number of chronic diseasese is the sum of the number of diagnosed above diseases

The OR of factors associated with depression and determined using multiple logistic regression analysis are shown in Table 2. Both men and women who did not practise health behaviours were more likely to have depressive symptoms than people who practised health behaviours (men, OR: 1.48, 95% CI: 1.31–1.68; women, OR: 1.42, 95% CI: 1.32–1.53). In addition, participants who had more chronic diseases, whose perceived health status was bad, or substantial stress were likely to be depressed.

**Table 2:** Results of factors associated with depression

Variables	Depression								
	Male					Female			
	OR	95% CI				OR	95% CI		
**Total**									
**Health behaviours**^**a**^									
Yes	1.00					1.00			
No	1.48	(1.31	–	1.68)		1.42	(1.32	–	1.53)
**Age (years)**									
19–29	1.00					1.00			
30–39	1.03	(0.85	–	1.26)		0.67	(0.58	–	0.77)
40–49	0.62	(0.50	–	0.76)		0.47	(0.41	–	0.55)
50–59	0.57	(0.46	–	0.71)		0.45	(0.39	–	0.53)
≥ 60	0.61	(0.49	–	0.76)		0.45	(0.38	–	0.54)
**Marital Status**									
Living with spouse	1.00					1.00			
Living without spouse	1.59	(1.43	–	1.77)		1.63	(1.52	–	1.75)
**Region**									
Metropolitan area	1.00					1.00			
Rural	0.92	(0.83	–	1.02)		0.92	(0.85	–	0.98)
**Occupational categories**^**b**^									
White	1.00					1.00			
Pink	1.35	(1.09	–	1.67)		0.99	(0.86	–	1.14)
Blue	1.21	(1.02	–	1.45)		0.88	(0.76	–	1.02)
Inoccupation	3.07	(2.56	–	3.69)		1.69	(1.50	–	1.91)
**Educational level**									
Middle school or less	1.65	(1.41	–	1.93)		1.77	(1.53	–	2.04)
High school	1.16	(1.01	–	1.33)		1.32	(1.18	–	1.48)
College or over	1.00					1.00			
**Household income**									
Low	2.21	(1.84	–	2.66)		1.89	(1.66	–	2.16)
Mid-low	1.22	(1.03	–	1.44)		1.32	(1.17	–	1.49)
Mid-high	0.99	(0.83	–	1.18)		1.09	(0.96	–	1.24)
High	1.00					1.00			
**Obesity Status (BMI)**^**d**^									
Underweight and Normal range	1.00					1.00			
Overweight	0.67	(0.59	–	0.75)		0.80	(0.73	–	0.87)
Obese	0.69	(0.62	–	0.78)		0.87	(0.80	–	0.94)
**The number of chronic diseases**^**e**^									
0	1.00					1.00			
1	1.25	(1.11	–	1.40)		1.26	(1.14	–	1.39)
≥ 2	1.56	(1.38	–	1.77)		1.47	(1.33	–	1.62)
**Perceived health status**									
Good	1.00					1.00			
Bad	4.16	(3.58	–	4.85)		2.75	(2.47	–	3.07)
**Perceived stress**									
Substantial	9.29	(8.41	–	10.26)		8.39	(7.83	–	8.98)
Less	1.00					1.00			

^a^Those classified as practising-health-behaviours group met all three conditions: not smoking (or 0 pack-year), not belonging to high-risk drinking group and walking for 30 min over 5 days per week

^b^Three groups (white, pink, blue) based on the International Standard Classification Occupations codes. Inoccupation group includes housewives

^c^*BMI* Body mass index/obesity status defined by BMI based on the 2018 Clinical Practice Guidelines for

Overweight and Obesity in Korea

^d^Chronic disease was defined diagnosed diseases: hypertension, diabetes mellitus, hyperlipidaemia and arthritis. The number of chronic diseasese is the sum of the number of diagnosed above diseases

Table 3 presents the association between individual criteria of health behaviours and depression stratified by gender. Among men, not walking frequently was the most influential factor associated with depression than other factors (not walking frequently, OR: 1.32, 95% CI: 1.20–1.46; smoking, OR: 1.17, 95% CI: 1.06–1.29; high-risk drinking, OR: 1.09, 95% CI: 0.97–1.23), while smoking was the most powerful factor in women (smoking, OR: 1.99, 95% CI: 1.75–2.26; high-risk drinking, OR: 1.43, 95% CI: 1.25–1.65; not walking frequently, OR: 1.25, 95% CI: 1.17–1.34). However, men who did not practise all of the health behaviours suggested in this study had the most powerful association with depression. In contrast, in women, only one factor, smoking, was more powerful than not practising all of the health behaviours.

**Table 3:** The results of subgroup anaylsis strafied by interesting variables*

Variables	Depression							
	Male					Female		
	OR	95% CI			OR	95% CI		
**High-risk drinking**^**a**^								
Yes	1.09	(0.97	–	1.23)	1.43	(1.25	–	1.65)
No	1.00				1.00			
**Smoking status**^**b**^								
Yes	1.17	(1.06	–	1.29)	1.99	(1.75	–	2.26)
No	1.00				1.00			
**Frequent walking**^**c**^								
Yes	1.00				1.00			
No	1.32	(1.20	–	1.46)	1.25	(1.17	–	1.34)

*Adjusted by variables including age, marital status, region, household income, job, educational status, pack year, BMI, physical activity, the number of chronic diseases, and perceived stress

^a^Defined as those who drink more than 1 day per week and have more than 5 shots (for women) or 7 shots (for men) Additionally adjusted by variables including smoking status and frequent walking

^b^Defined as those who do not smoke currently and pack year is ‘0’, and additionally adjusted by variables including high-risk drinking and frequent walking

^c^Defined as those who walk for 30 min over per day and over 5 days per week. Additionally adjusted by variables including high risk drinking and smoking status

**Fig. 2:**
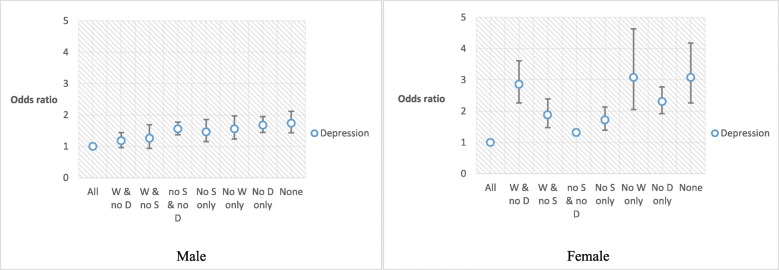
The results of subgroup analysis stratified by interesting variables

The combination of each factor of health behaviours and its relation to depression is shown in Fig. 2. As can be seen from the figure, fewer the health behaviours practised, greater was the relation to depression. Moreover, participants who never practised health behaviours were the most likely to have depressive symptoms (men, OR: 1.69, 95% CI: 1.38–2.07; women, OR: 3.08, 95% CI: 2.27–4.19) (Table 2 in Supplement). The most influential factor among men and women in Fig. 1 had a thread of connection with Table 3.

The subgroup analysis stratified by independent variables is represented in Table 3 of Supplement. We examined variables related to health behaviours and confirmed the association of depression in most not-practising-health-behaviours groups compared to practising-health-behaviours groups.

## Discussion

The present study was designed to determine the association between health behaviours and depression in a large population-based sample. The results showed that not practising health behaviours was significantly associated with depression among Korean adults. The ORs of association between health behaviours and depression were higher in men than in women.

To confirm the most influential factor of health behaviours, we analysed the relationship between each factor and depression. Men and women had different results. Not walking frequently was the most influential factor in men, and smoking was the most associated with depression in women. In other words, men who do not practise frequent physical activities are more associated with depression than those who do, and women who smoke are more likely to have depressive symptoms than those who are not smokers. However, men who did not practise all of the health behaviours suggested in this study, had a more powerful association with depression than only the not-walking-frequently group.

We further analysed the relationship between health behaviours and depression. There were different results for men and women, which could indicate different influences. However, in general, it has been shown that higher the number of not-practising-health-behaviours factors, the greater the association with depression. It means that practising good health behaviours is related to a decrease in depression.

Living without a spouse, having no jobs at that time, low educational status, low household income, comorbidity, perceived health status, and substantial perceived stress were associated with depression in this study. Further analysis showed that the participants, especially men, with worse health factors had a greater association with depression when they did not practise health behaviours.

Moreover, participants who had more chronic diseases and did not practise health behaviours were more associated with depression. According to several studies that explained the relationship between hypertension and depression, hypertension was significantly associated with depression because of abnormal circadian blood pressure [25], increasing sympathetic nervous system activity [26, 27], genetic factors [28], etc. Likewise, other chronic diseases included in this study were associated with depression [29–31].

Consistent with past studies, health behaviours selected in this study were confirmed to have a relationship with depression. First, smoking increases sympathetic nervous system activity [32] and could have an effect on depression. Second, physical inactivity is a leading cause of chronic disease [33], and participants in this study who had chronic diseases and did not practice health behaviours were vulnerable to depression [34–37]. Additionally, a meta-analysis study found that exercise is an effective mediation for depression because it is related to expansion of brain capacity [38]. Lastly, a study considering people’s drinking status, intensity, and frequency found that alcohol consumption is related to depression [39].

Some studies showed that interventions for reducing depression varied by subjects and objectives. Improving lifestyle, in particular, has been proven by previous studies to be effective in treating depression. Physical activity [40] intervention including walking [41] is effective in reducing depression. In addition, management of recreational substances such as alcohol, cigarettes, and caffeine is effective in the treatment of depression [42].

The present study has several strengths. We used a large and nationally representative database [21] to determine lifestyle factors associated with depression. In addition, we used the PHQ-9, which is a reliable and valid tool for screening depressive patients than a self-reported questionnaire [43]. To our knowledge, this is the first study to determine the relationship between a health behaviours and depression, using the CHS data. In addition, we found that the most influential health behaviour factors in depression were different for men and women.

However, this study has some limitations. First, because this was a cross-sectional study, we could not prove whether health behaviours were a cause or a consequence of depression. Second, factors of health behaviour were self-reported; participants had to respond by relying on their memory, and the responses might not be accurate. Especially, according to previous research, South Korean women tend to hide their real smoking history [44]. This may have affected our results. Third, other health behaviours or environmental factors may also have a hand in the development of depression. However, we analysed the main results including other possible factors as covariates. Fourth, we did not consider the intensity and duration of walking. Lastly, we could not exclude exact patients who had depression, because of the lack of a questionnaire in the CHS. However, we eliminated participants who had a history of expert consultation for sadness or hopelessness that continued more than two weeks in the previous year.

## Conclusion

This study found that influential factors of depression are different for men and women. In addition, participants who did not practise any health behaviours were the most likely to have depressive symptoms. Efforts are underway to manage depression in various ways and according to individual differences. As previously mentioned, depression is a significant global health concern and requires social attention and consideration. Therefore, it is necessary to manage depression through interventional methods customised to gender characteristics. Additionally, national-level policies are needed to encourage steps to improve personal lifestyles, including practising health behaviours.

## Supplementary information

**Additional file 1: Table S1.** The results of comparison of model selection statistics among a candidate models. **Table S2.** The results of subgroup analysis stratified by interesting variables*. **Table S3.** The results of subgroup analysis stratified by interesting variables.

## Data Availability

The CHS data are openly available at https://chs.cdc.go.kr/chs/index.do by submitting a written statement and data utilization plan.

## Abbreviations

OR: Odds ratio
CI: Confidence interval
OECD: Organization for Economic Co-operation and Development
PHQ-9: Patient Health Questionnaire-9
CHS: Community Health Survey
VIF: Variance inflation factor
